# Seasonal agricultural workers' personal well-being and preventive behaviors about Covid- 19 in Turkey

**DOI:** 10.1186/s12889-023-15024-z

**Published:** 2023-01-14

**Authors:** Sevda Yaman, Mahmut Kilic

**Affiliations:** 1 Akdagmadeni Health School, Yozgat Bozok University, Yozgat, Turkey; 2 Department of Public Health, Faculty of Medicine, Yozgat Bozok University, Yozgat, Turkey

**Keywords:** Agricultural workers, Covid-19, Linear regression, Measures, Personal wellbeing

## Abstract

**Background:**

Seasonal agricultural workers working and living in inappropriate sanitary conditions are at great risk for public health. This study aimed to determine the relationships between the sociodemographic variables and life satisfaction of seasonal agricultural workers, and their knowledge, risk perception, and protective behaviors about the COVID-19 pandemic.

**Methods:**

This is a cross-sectional study, that included agricultural workers who are 18 years of age or older and worked seasonally in Yozgat, Turkey, during the period between August 2020 and October 2020. The well-being level was measured using the Personal Wellbeing Index-Adult form (PWIA). The data were collected using the face-to-face survey method and with 739 workers who voluntarily participated in the research.

**Results:**

All participants disclosed having insufficient information about Covid-19 and indicated their peers and television as their sources of information. The vast majority of the workers stated that they complied with the mask mandates, social distancing, and hand hygiene. No correlations were found between knowledge, attitudes, and behaviors about Covid-19 and the level of wellbeing. The mean PWIA score of the workers was low (53.7) while they were mostly satisfied with their personal relationships (96.6) and health (76.1). The multivariable linear regression analysis revealed that being male (β = 0.245) and not having an ongoing health issue (β = 0.689) were associated with more PWIA; on the other hand, having more children (β = -0.52) was related to less PWIA.

**Conclusions:**

The well-being level of seasonal workers was lower while it was not associated with knowledge, attitudes, and behaviors about Covid-19.

## Introduction

Seasonal agricultural labor involves ongoing or migratory seasonal labor done by the citizens of a country or immigrants in a country in compensation for pay, daily wage, or pay in kind for their work in any stage of agricultural activities such as sowing, growing, pest control, and harvest in the agricultural land of their own or another person(s) [1]. Seasonal migratory agricultural workers usually go to places with job opportunities to work with their family members, supplies, and tools. Therefore, they stay in places that are allocated by their employers. The workers try to have shelter and, thus, hold onto life in those allocated locations.

The Covid-19 epidemic has spread around the world. In a report published by the International Labour Organization (ILO) on 7 April 2020, those working in the agricultural sector were considered to be among the "sectors at the greatest risk" during the Covid-19 pandemic [2]. The workers in the agriculture sector are a disadvantaged population in terms of both their health and wellbeing due to being the third main sector in Turkey, demanding and dangerous jobs, and having unregistered employment in seasonal agricultural works [3, 4].

The concept of "wellbeing" is frequently used in the literature and included in the "definition of health" proposed by the World Health Organization (WHO). It includes the concepts of quality of life, happiness, and life satisfaction [5]. Wellbeing is responsible for a range of social, economic, and political factors and is at high levels in developed countries. It is negatively affected especially in low-income populations during the Covid-19 pandemic [6, 7].

The lower level of health and wellbeing of disadvantaged groups in society is a well-known fact. Seasonal agricultural workers face multilayers of insecurity such as sociocultural status, education, and language problems. Agricultural workers are among the disadvantaged groups in society. Their health status, access to health services, knowledge, attitudes, and behaviors about diseases, and wellbeing are at lower levels than those of the overall population [8, 9]. They are prone to endemic diseases while having varying lifestyles [10]. In a study conducted in Pennsylvania in the United States (USA), it was determined that chronic diseases such as heart disease, stroke, asthma, diabetes, obesity and hypertension are seen at high rates in seasonal agricultural workers due to low income, limited time to prepare meals and malnutrition. Workers have a high incidence of illness and premature death due to unsuitable housing and working conditions, inadequate and unbalanced nutrition, accidents and injuries, pesticides, exposure to extreme heat and cold, and lack of access to health services. For this reason, seasonal agricultural workers are included in the special risk group in agricultural societies. The problems experienced affect seasonal agricultural worker women more. Inadequate hygiene conditions, low socio-economic level, marriage at a young age and adolescent pregnancies, inability to access prenatal, postnatal and postnatal health services in agricultural areas increase the health risks of mother and baby. Differences such as gender, age, education, marital status, income, and health status affect subjective well-being [8]. The Personal Wellbeing Index (PWI) measures individuals' self-satisfaction within this context. It has been applied in many countries to different samples [8, 9].

Considering the risks, they face depending on the quality of the work and improper living conditions, health issues, hardships in access to basic human rights (health, education, and social services), the evaluation of the attitudes and behaviors of seasonal agricultural workers during the COVID-19 pandemic will help better understand the public awareness of the pandemic, handle the gaps in knowledge, strengthen the ongoing preventive measures, and provide better insight.

The present study aimed to determine of sociodemographic characteristics and the relationship between the life satisfaction of seasonal agricultural workers and knowledge, risk perception, and protective behaviors about the COVID-19 pandemic.

## Methods

### Research method

This is a cross-sectional study.

### The universe and sample of the study

This study included seasonal agricultural workers. The sample consisted of agricultural workers aged 18 and over who worked seasonally in Yozgat, Turkey, during the period between August 2020 and October 2020. Since the total number of the seasonal agricultural workers was unknown, the sample size was calculated to be 384 for unknown population size, a significance level of *p* = 0.5, ɑ = 0.05, and deviation of d = 0.05. The data were collected by the researchers using the face-to-face survey method and from 739 individuals after obtaining their voluntary consent.

### Data collection tools

The data were collected using the socio-demographic form that was created by the researchers, the Covid-19 survey, and the Personal Wellbeing Index-Adult (PWI-A) scale.

#### Covid-19 survey

The Covid-19 survey that is prepared by the authors includes 20 questions about knowledge, attitudes, and behaviors regarding the transmission of Covid-19, related protection measures, and control methods. The covid-19 survey is not a scale. There are 3 knowledge questions about Covid 19, and the answer options for each question were told to the subjects. “How has Covid-19 transmitted?” 10 options, “What are the symptoms of the disease?” 5 options, “What precautions can be taken against the disease?” 13 options. There are 6 attitude questions about Covid 19, and the answers are Likert-type. “Do you find the measures taken at your workplace during the Covid-19 period sufficient?” etc. There are 6 behavioral questions about protection from Covid 19, and the answers are Likert type. “Can you comply with the social distance rule of 1.5–2 m between people?” etc.

#### Personal Wellbeing Index – Adult (PWI-A)

The scale is used to measure the personal well-being of individuals over the age of 18 and includes 7 items about satisfaction, with each item corresponding to a quality-of-life domain comprising standards of living, health, achievements in life, relationships, security, community-connectedness, and future security. A single measure of life satisfaction is obtained by calculating the mean score of the seven domains. The PWIA is complemented by an independent item that measures satisfaction with life as a whole, namely overall life satisfaction (OLS). This item is not a part of the scale but is used to confirm the psychometric properties of the scale. The question of satisfaction with religion or spirituality is optional and its inclusion in the total score of the scale is left to the discretion of the researchers [11]. In the present study, the question regarding satisfaction with religion or spirituality was not included in the total score of the scale, with the thought that people might be affected by the interviewer and not fully report their actual thoughts. The results of the study revealed that the mean satisfaction with religion or spirituality (96.7%) was much higher than the general mean (53.7%). Participants rate their level of satisfaction on an 11-point scale from 0 (not at all satisfied) to 10 (completely satisfied). The scores were converted to a standard range of 0–100 to compare the results to those obtained in previous studies (International Wellbeing Group 2006). The internal consistency Cronbach Alpha value of the scale was 0.81, which was deemed sufficient.

#### Data analysis

The data were analyzed using IBM SPSS Statistics Standard Concurrent User V 25, Authorization Code: e31d836848b0a60e5756. The Student's t-test, ANOVA, and Post-hoc Bonferroni test were used to analyze the differences between the mean PWIA values of the groups. The statistically significant independent variables as revealed by the univariate tests were analyzed using the multivariable linear regression (LR) backward model. As categorical and ordinal variables, gender, permanent residence, smoking status, having an ongoing health problem, educational levels of the participants and their spouses were included in the LR analysis as dummy variables. Education level was 4 groups, and high school and over was taken as the reference category. Table 4 shows the statistically significant variables (*p* < 0.05).

## Results

### Description of the sample

The mean age of the participants was 32.9 ± 8.2 (min:18, max:55) and 49.5% of the participants were female, 45.3% were illiterate, 97.8% were married, and 68.7% had 3 or more children. The participants stated having no social security but not having problems in access to health services. Among the participants, 85.5% stated having permanent residence in central districts and 17.5% stated having an ongoing health problem.

### Association between sociodemographic variables and well-being

The univariate tests revealed that males, those who were in the 18–39 age group, those with an elementary-level education or above, those who have illiterate spouses, those who permanently live in urban areas, smokers, and those who do not have an ongoing health problem had higher PWIA scores (Table 1).

**Table 1:** PWIA mean values by socio-demographic variables

**Socio-demographic variables**			**PWIA**		**t/F**	**Overall Life**		**t/F**
	**Count**	**Col. %**	**Mean**	**95% C.I**	**p**	**Mean**	**95% C.I**	**p**
**Gender**								
Male	373	50.5	56.7	54.9–58.4	t = 4.84	44.9	42.1–47.8	t = 4.76
Female	366	49.5	50.7	49.1–52.4	*p* < .001	34.6	31.5–37.8	*p* < .001
**Age groups **^**a**^								
18–24	129	17.5	56.6	53.9–59.4	F = 4.21	43.6	38.3–49.0	F = 3.06
25–29	133	18.0	54.0	51.2–56.7	*p* < .001	38.9	34.0–43.9	*p* < .01
30–34	146	19.8	55.6	53.0–58.3		42.5	37.8–47.3	
35–39	185	25.0	54.1	51.6–56.6		41.8	37.6–46.1	
40–44	74	10.0	50.1	45.8–54.4		34.3	27.3–41.3	
≥ 45	72	9.7	47.0	42.8–51.1		29.7	22.7–36.7	
**Education Level**								
Illiterate	335	45.3	51.1	49.4–52.9	t = 3.86	35.5	32.2–38.8	t = 3.64
Elementary school and higher	404	54.7	55.9	54.2–57.6	*p* < .001	43.4	40.6–46.3	*p* < .001
**Education Level of the Spouse**								
Illiterate	336	45.5	56.5	54.6–58.3	t = 4.02	44.5	41.5–47.5	t = 3.95
Elementary school or higher	403	54.5	51.5	49.9–53.1	*p* < .001	36.0	33.0–39.0	*p* < .001
**Number of children**								
None	34	4.6	56.3	50.9–61.8	F = 2.86	47.9	37.2–58.7	F = 2.29
1	77	10.4	57.6	53.7–61.6	*p* < .014	46.2	39.1–53.4	*p* < .05
2	120	16.2	54.5	51.7–57.4		39.6	34.2–44.9	
3	144	19.5	55.1	52.5–57.6		40.4	35.9–44.9	
4	121	16.4	54.3	51.1–57.5		41.5	35.8–47.2	
≥ 5	243	32.9	50.7	48.5–52.9		35.6	31.9–39.3	
**Permanent Residence**								
Rural Area	63	8.5	47.9	43.7–52.1	t = 2.88	31.3	24.2–38.4	t = 2.39
Urban	676	91.5	54.3	53.0–55.6	p < .01	40.6	38.4–42.9	p < .02
**Smoking status**								
Non-smoker	427	57.8	51.2	49.6–52.7	t = 4.93	35.5	32.6–38.4	t = 4.72
Smoker	312	42.2	57.3	55.4–59.1	*p* < .001	45.8	42.7–48.9	*p* < .001
**Presence of ongoing health issues**								
No	610	82.5	59.2	58.2–60.3	t = 27.05	48.2	46.2–50.3	t = 21.02
Yes	129	17.5	27.8	27.2–28.3	*p* < .001	.2	-0.2–0.7	*p* < .001
**Total**	**739**	**100.0**	**53.7**	52.5–55.0		**39.8**	37.7–42.0	

Student's t-test was used for variables with 2 groups, and the ANOVA test for those with 3 or more groups

*PWIA* Personal Wellbeing Index-Adult

^**a**^ Post hoc Bonferroni test: 45 + group is different from the 18–24, 30–34, and 35–39 age groups

According to the PWIA score sub-items, the participants were mostly satisfied with their personal relationships (96.6) and health (76.1) while they were least satisfied with their feeling of security (34.0) and future security (34.0). Satisfaction with religion or spirituality was very high (96.6). Moreover, the living standards, the feeling of security, future security, total satisfaction, and overall life satisfaction scores were higher in men than in women (Table 2).

**Table 2:** Personal Wellbeing Index-Adult scores by gender

	**Gender**					
	**Male**		**Female**		**Total**	
	**Mean**	**95% C.I**	**Mean**	**95% C.I**	**Mean**	**95% C.I**
Satisfaction with life standards^**^	44.9	42.1–47.8	34.6	31.5–37.8	39.8	37.7–42.0
Satisfaction with health state	77.0	73.4–80.7	75.1	71.2–79	76.1	73.4–78.7
Satisfaction with life-achievements	44.1	42.6–45.7	44.3	42.7–45.9	44.2	43.1–45.3
Satisfaction with personal relationships	96.7	95.9–97.5	96.6	95.7–97.4	96.6	96.0–97.2
Satisfaction with the level of confidence **	40.5	37.8–43.3	27.3	24.7–29.8	34.0	32.0–35.9
Satisfaction with community	52.9	50.8–54.9	50.1	48.1–52	51.5	50.1–52.9
Satisfaction with future security **	40.5	37.8–43.3	27.3	24.7–29.8	34.0	32.0–35.9
Total PWIA ^**^	56.7	54.9–58.4	50.7	49.1–52.4	53.7	52.5–55.0
Overall life satisfaction **	44.9	42.1–47.8	34.6	31.5–37.8	39.8	37.7–42.0
Satisfaction with religion/spirituality	96.6	96.1–97.1	96.8	96.3–97.3	96.7	96.4–97.0

*PWIA* Personal Wellbeing Index-Adult

^**^ Significant at the 0.01 level (2-tailed)

Using linear regression, the multivariable analysis of the variables that were related to the PWIA or had different mean values revealed that being male (β = 0.245) and not having an ongoing health issue (β = 0.689) were associated with more PWIA; on the other hand, having more children (β = -0.52) was related to less PWIA. These 3 variables explain 52.1% of the change in well-being (Table 3).

**Table 3:** Analysis of the factors affecting the PWIA score using the linear regression backward model

	Unstandardized Coefficients		Standardized Coefficients	t	P	95.0% Confidence Interval for B	
	B	Std. Error	β			Lower Bound	Upper Bound
(Constant)	23.928	2.391		10.007	.000	19.234	28.623
Gender = Male	8.297	2.201	.245	3.769	.000	3.976	12.619
Education Level = Illiterate	3.708	2.212	.109	1.677	.094	-.634	8.050
Number of children	-.374	.187	-.052	-2.006	.045	-.741	-.008
Ongoing health issue = No	30.735	1.158	.689	26.534	.000	28.461	33.009

Independent variables: Dummy variables, gender, permanent residence, smoking, and presence of an ongoing health problem, education level and spouse education level; continuous variables, age, and the number of children. *PWIA* Personal Wellbeing Index-Adult

R = 0.724, Adj. R2 = 0.521

### Association between covid-related variables and well-being

All participants stated having insufficient information about Covid-19 and having their friends (100.0%), television (93.6%), and the Internet/social media (9.2%) as their sources of information. All participants had accurate knowledge about the transmission of Covid-19 by sneezing, coughing, direct contact with people, inanimate surfaces, and through the air. However, 31.8% of the participants stated that Covid-19 is transmitted by touching or eating foodstuffs, 23.7% stated that it is transmitted by contact with animals, and 5.5% stated that it is transmitted through water. All of the participants correctly identified the symptoms of Covid-19 as fever, cough, sore throat, and shortness of breath. All participants correctly identified the appropriate behaviors comprising avoiding physical contact, washing hands, wearing masks, avoiding contact with eyes with dirty or unclean hands, covering the mouth and nose during coughing-sneezing, consuming vegetables-fruits and protein-rich foods, drinking plenty of fluids, and going to health institutions when they have symptoms. However, 23.7% of the participants incorrectly identified contact with animals as a necessary measure against Covid-19. Almost all participants (96.5%) correctly indicated individuals over the age of 65 years, cancer patients, those with a weak immune system, those with hypertension, diabetes, and lung disease, pregnant women, and smokers as the groups that face the greatest risk in terms of Covid-19. On the other hand, 96.5% incorrectly stated that alcohol users, drug users, infants, and children were in the highest risk group (Table 4).

**Table 4:** Distribution of knowledge, attitudes, and behaviors about Covid-19 by PWIA’s mean

**Answer “Yes”**				PWIA		t/F
		Count	%	Mean	**95% C.I**	p
Infection—Through eating	No	504	68.2	53.8	52.3–55.2	t = .067
	Yes	235	31.8	53.7	51.5–55.9	P = .947
Infection—Touching foodstuffs	No	504	68.2	53.8	52.3–55.2	t = .067
	Yes	235	31.8	53.7	51.5–55.9	.947
Infection—Touching animals	No	564	76.3	53.6	52.2–55.0	t = .460
	Yes	175	23.7	54.3	51.8–56.7	*p* = .646
Infection—via Water	No	698	94.5	53.7	52.5–55.0	t = .050
	Yes	41	5.5	53.9	48.9–58.8	p = .960
Knowledge—Incubation period	1 -14 days	709	95.9	53.6	52.4–54.9	t = .793
	15 -20 days	30	4.1	56.1	48.6–63.6	*p* = .428
Attitudes—Measures taken at residences and workplaces	I find it very inadequate	332	44.9	53.8	52.0–55.6	F = .109
	I find it slightly adequate	320	43.3	53.9	52.0–55.8	*p* = .897
	I find it very adequate	87	11.8	53.0	49.0–56.9	
Attitudes—Fear of contracting Covid-19	I do not fear whatsoever	.0	.0	.0	.0	F = 1.849
	I fear slightly	38	5.1	49.8	43.5–56.0	*p* = .137
	I fear moderately	157	21.2	55.9	53.1–58.6	
	I fear quite much	56	7.6	55.3	50.3–60.2	
	I fear very much	488	66.0	53.2	51.7–54.6	
Attitudes—Measures taken by colleagues	I find it very inadequate	332	44.9	53.8	52.0–55.6	F = 1.694
	I fear slightly	183	24.8	52.5	50.1–55.0	*p* = .167
	I fear moderately	178	24.1	55.7	53.1–58.3	
	I fear quite much	46	6.2)	50.5	44.8–56.1	
	I fear very much	.0	.0	.0	.0	
Attitudes—Can you get infected?	No	.0	.0	.0	.0	
	Yes, possibly	407	55.1	53.7	52.0–55.4	t = .069
	Yes, highly probable	332	44.9	53.8	52.0–55.6	*p* = .945
Attitudes—Being vaccinated if one is available	Definitely no	.0	.0	.0	.0	
	Maybe	53	7.2	55.4	50.6–60.2	t = .725
	I will definitely be vaccinated	686	92.8	53.6	52.3–54.9	*p* = .469
	Undecided	.0	.0	.0	.0	
Attitudes—Will the measures protect from the virus?	I very much believe so	44	6.0	53.7	48.4–58.9	F = .020
	I believe so	135	18.3	53.5	50.5–56.4	*p* = .980
	I partially believe so	560	75.8	53.8	52.4–55.2	
	I do not believe so	.0	.0	.0	.0	
Behaviors- Obeying social distancing	I do not obey whatsoever	.0	.0	.0	.0	
	I sometimes obey	66	8.9	54.3	49.8–58.7	t = .264
	I generally obey	673	91.1	53.7	52.4–55.0	*p* = .792
	I always obey	.0	.0	.0	.0	
Behaviors- Wearing a mask	I always wear a mask	739	100.0	53.7	52.5–55.0	
Behaviors- Wearing protective gloves	I never wear protective gloves	739	100.0	53.7	52.5–55.0	
Behaviors- Washing hands	I do not wash my hands	.0	.0	.0	.0	
	Sometimes	111	15.0	54.8	51.6–57.9	t = .699
	Generally	628	85.0	53.6	52.2–54.9	*p* = .485
	Always	.0	.0	.0	.0	
Behaviors—Using disinfectants—cologne	I never use	.0	.0	.0	.0	
	Sometimes	690	93.4	53.8	52.5–55.1	t = .478
	Generally	49	6.6	52.6	47.8–57.5	*p* = .634
	Always	.0	.0	.0	.0	
Behaviors – Following a healthy diet	I do not follow a healthy diet	33	4.5	54.0	48.7–59.4	F = 1.536
	Sometimes	623	84.3	54.1	52.8–55.5	*p* = .216
	Generally	83	11.2	50.7	47.0–54.4	
	Always	.0	.0	.0	.0	
Behaviors- Changes in dietary habits- Drinking plenty of water	No	699	94.6	53.7	52.5–55.0	t = .064
	Yes	40	5.4	53.6	48.3–58.9	*p* = .949
Behaviors—Limiting social relationships	Never	122	16.5	54.6	51.6–57.7	t = .641
	Yes, partially	617	83.5	53.6	52.2–54.9	*p* = .522
	Yes, generally	.0	.0	.0	.0	
Access to protective materials	Never	.0	.0	.0	.0	
	I hardly have access to protective materials	690	93.4	53.8	52.5–55.1	t = .476
	I have adequate access to protective materials	49	6.6	52.6	47.8–57.5	*p* = .634
Purchasing protective materials	I can never purchase protective materials	.0	.0	.0	.0	
	I can hardly purchase protective materials	690	93.4	53.8	52.5–55.1	t = .476
	I can adequately purchase protective materials	49	6.6	52.6	47.8–57.5	*p* = .634
Did the expenses increase during the Covid-19 pandemic?	No, it did not increase	.0	.0	.0	.0	
	Yes, slightly increased	690	93.4	53.8	52.5–55.1	t = .476
	Yes, substantially increased	49	6.6	52.6	47.8–57.5	*p* = .634
	Decreased	.0	.0	.0	.0	
**Total**		**739**	**100.0**	**53.7**	**52.5–55.0**	

The cases where 95% or more of the questions were answered yes were not included in the table

Student's t-test was used for variables with 2 groups, and the ANOVA test for those with 3 or more groups

*PWIA* Personal Wellbeing Index-Adult

Of the participants, 95.9% knew the incubation period of Covid-19. Among the participants, 44.9% found the measures that were taken in their places of work or residence inadequate while 11.8% of the participants found them quite sufficient; 69.7% of the participants find the measures taken by their colleagues as insufficient or somewhat sufficient; 73.6% of the participants indicated being very afraid of the transmission of the disease while 44.9% of the participants stated that the virus will eventually infect them or members of their family. Among the participants, 24.3% believed that the measures they have taken would protect them from the virus and 92.8% stated that they would be vaccinated if a vaccine were available (Table 4).

All participants stated that they always wear masks to protect themselves from Covid-19, but they never wear gloves, do not consume protective food, and take preventive medicine. Of all participants, 91.1% generally followed the social distancing measures; 85% generally washed their hands; 83.5% partially limited their social relations; and. Of the workers, 93.4% stated that they hardly had access to or could buy protective materials. All participants stated that their expenses increased to a certain degree during this period. The results revealed that the knowledge, attitudes, and behaviors of the workers about Covid-19 were not significantly related to their well-being scores (Table 4).

## Discussion

In the present study, the life satisfaction of seasonal agricultural workers and the workers’ knowledge, risk perception, and protective behaviors about the COVID-19 pandemic, and the relationship between them were examined. The participants had the highest level of well-being in personal relationships (96.6 points) while having the lowest well-being in the feeling of security (34.0 points) and future security (34.0 points). It is an expected finding that seasonal agricultural workers, who come from similar regions for work, live collectively in a tent environment, and have similar education levels, have good personal relations. However, having social security and health problems, having many children, and not having a regular income can prevent them from feeling safe.

Women make up half of the workforce in agriculture [12]. Although women constitute half of the workers participating in our research, the well-being of women was found to be lower than that of men. While women do income-generating jobs for financial security for themselves and their households, they have to balance their other responsibilities. The tiring agricultural work and the problems with housing opportunities make women even more disadvantaged. Similarly, in a study conducted in rural China, women have lower PWI than men [13]. Our findings can also bring to mind the concept of gender inequality that exists in our country and similar societies.

It is an expected finding in our study that there is a significant relationship between education level and PWI. Educated individuals can be considered to be more advantageous in overcoming difficult tasks and having positive life experiences by feeling a sense of personal importance. Seasonal agricultural workers have a lower education level than the general population due to their nomadic life and low income.

Another remarkable finding in our research is that the increase in the number of children reduces well-being. The family is primarily responsible for the education and upbringing of children. However, problems such as the economic structure in the family, the fact that the mother is also working, the agricultural workers having more children, and the disruption of the educational life of the children during the pandemic period may have negatively affected their well-being. As a matter of fact, agricultural workers have more children than the normal population. In studies conducted on the general population in South Australia and Lithuania, the increase in the number of children negatively affects well-being [11, 14].

It is a reality accepted by everyone that health is the most valuable thing in human life. In such a case, it is inevitable that health status will be an important component in determining the level of welfare. It is known that studies conducted in many different countries put their health and work conditions first among the factors that most affect people's living standards [15]. In our study, the well-being of those who did not have a current health problem was found to be high. Our finding is in line with studies across and rural China and in Lithuania [14, 16, 17]. Agricultural work is also a profession that has problems such as drought, epidemics, market fluctuations, geographical and social isolation and can strain welfare. It is also associated with serious health problems, life traumas, and poor social connections [18]. However, different results are reported even from developed countries on the health risks that affect the welfare of agricultural workers. Although health is one of the most important determinants of well-being, agricultural workers make insufficient use of health services in most regions of the world [19]. In a study conducted in Canada, it was determined that migrant agricultural workers could not be adequately protected during the pandemic period, and a low level of welfare was found in the workers in this process [20]. In our study, it was found that employees were not sufficiently protected from covid 19 and had low welfare.

In the study conducted by statistics using the life satisfaction dataset in Turkey, those living in the eastern provinces were determined to have the lowest level of subjective well-being [3]. All participants in this study were from Şanlı Urfa, one of the provinces in the southeastern region of Turkey. In the pre-pandemic period, the PWI scores of those in countries such as Australia, the Netherlands, and Austria were around 75 points on a 0–100-point scale while they were lower in developing countries [21]. During the pandemic, a decrease in the well-being score was also observed in Australia [22]. This is also true for individuals over the age of 65 in Turkey [23].

Seasonal agricultural workers are a disadvantaged group in many aspects such as their inability to participate in social activities due to their intense work schedule and low income and their well-being during the pandemic process is a neglected issue in Turkey. No studies on the knowledge, attitudes, and behaviors of agricultural workers about Covid-19 were found in the literature. The relationship between the well-being levels of the participants and their knowledge, attitudes, and behaviors about Covid-19 was examined and no statistically significant relationships were found. We expect this study will contribute to raising awareness of the health and well-being levels of seasonal agricultural workers.

Almost all participants believed that their knowledge of Covid-19 was insufficient in addition to having low well-being. Peers were their leading information source, followed by television and the Internet (Table 4). According to an online study in Turkey, the news and developments regarding the Covid-19 outbreak were obtained from similar sources [7]; however, the ratio of having peers as the information source was found to be higher in our study. This was associated with the lack of electricity in some tent areas and the high social interactions due to the collective life in those areas.

All participants correctly knew about the transmission of Covid-19 by contact and droplets (Table 4). The ratio of correct knowledge of transmission through direct contact and droplet in the Indian community has been reported to be 91.7% [24]. Our results are close to those found by Kılıç et al. (2021) in the same province and the online study conducted by Şirin et al. (2020) one month after the first case in Turkey [25, 26]. Regarding the symptoms of Covid-19, all participants correctly identified fever, cough, sore throat, and shortness of breath as symptoms while 95.3% correctly identified diarrhea as a symptom (Table 4). The ratio of correctly identifying the first three symptoms was almost 100 (both in Turkey and India [24, 26]. In a study conducted in Nigeria, the ratios of correct knowledge of the transmission route and symptoms of the disease are similar [27]. On the other hand, the agricultural workers in Turkey knew more about the ways of protection and risk groups than the Indian sample [24]. The knowledge and behavior of farm workers in North Carolina about covid 19 are similar to our findings [28].

Almost all workers correctly identified the high-risk groups in terms of Covid-19. On the other hand, 96.5% incorrectly stated that alcohol consumers, drug users, infants, and children were in the highest risk group (Table 4). Incorrect information about the transmission route and risk groups may be related to the low education levels of the participants, as well as the dissemination of disinformation at the beginning of the pandemic.

Attitudes about COVID-19 have different origins such as stereotypes about similar viral diseases, the government, social media, the Internet, previous personal experiences, and medical sources. The accuracy of these beliefs can lead to different prevention-related behaviors and differ by population. In many cases, the lack of knowledge, misinformation, or misunderstanding of medical sciences can pose a risk. The examination of the attitudes about Covid-19 revealed that most participants find the precautions in their places of work and residence and the precautions taken by their colleagues insufficient. The participants were very afraid of contracting the disease themselves or their family members and expressed disbelief in protective measures (Table 4). The high ratio of willingness to be vaccinated when a vaccine is available was close to 100%, which can be regarded as a positive attitude.

Although most of the workers stated that they tried to adapt to social isolation and wash their hands, they said that they had difficulty in reaching protective materials. They stated that they could not meet these expenses due to their low financial income. This situation is similar not only for agricultural workers but also in the general Turkish society [7, 25, 26, 29]. In China, it was emphasized that the most frequently emphasized way of protection was hygiene, and an increase in hygiene practices was observed. The fact that our hygiene practices are higher than countries such as India and Egypt during the Pandemic process may be due to the importance given by the administrations and the media to the issue as well as cultural differences. [30–33].

About one-fifth of the total population in Turkey works in the agricultural sector. Seasonal agricultural workers constitute the majority of informal workers. However, there is no data system for the seasonal migrant workforce of the agricultural sector [34]. Our research is important in terms of being the first source to enter the literature on these employees. Our research includes important findings both about the pandemic process and about wellbeing in this population. Our findings can also be used as a source for comparisons in terms of occupational health, which is an important public health problem.

### Limitations

This study is limited to seasonal workers working in central Yozgat, Yerköy, Sorgun, and Akdağmadeni. While the survey data was being collected, some employees were not in the tent environment, and the workers who went to the city center to meet their daily and basic needs did not participate in the survey. In addition, clear information about the number of employees could not be obtained because they were not officially registered. Since the working environment was not suitable for pregnant and postpartum women, this group was not included among the participants. Some employees were excluded from our research because they spoke a different language.

## Conclusion and recommendations

This study was conducted when the number of COVID-19 pandemic cases in Turkey was very high. Although it is limited in terms of its representativeness and generalizability, the study revealed the current situation, awareness, attitudes, and behaviors about the pandemic, and analyzed the relationship between these variables and the PWI of agricultural workers. Although agricultural workers have some negative attitudes about the pandemic, they are knowledgeable about the symptoms, findings, and precautions, which are insistently emphasized by television, social media, and the social environment. This may be an indicator of seasonal agricultural workers' trust in the media and social environment. In this regard, the information provided by social communication channels gains importance.

The high number of children, the fact that the workers are women, the low level of education and the negative effects of existing health problems on their well-being indicate that education and health policies should be rearranged for disadvantaged groups. The low PWI of agricultural workers who have to live in difficult conditions shows the need for them to attain safe and healthy living standards. Considering the current situation, it can be suggested that local governments and non-governmental organizations come together and develop projects. During the pandemic period, it may be suggested by local governments to provide hygiene materials to this population, and methods can be planned to facilitate their access to health services. Health education should be done by professional teams by observing on-site, and social activities should be planned to increase their well-being.

## Data Availability

The datasets generated and/or analyzed during the current study are not publicly available due to limitations of ethical approval involving the participants’ data and anonymity but are available from the corresponding author on reasonable request.

## References

[1] Sarper S. İş Hukuku, Beta Yayınları, İstanbul, 2009;231. ISBN: 9786052427682

[2] Ilo. (2020). Ilo Gözlem 2nci Baskı: Covid-19 Ve Çalışma Yaşamı. 7 Nisan 2020. https://www.İlo.Org/Wcmsp5/Groups/Public/---Europe/---Ro-Geneva/---İlo-Ankara/Documents/Briefingnote/Wcms_741784.Pdf.

[3] Çağlar A (2020). İllerin Yaşam Kalitesi: Türkiye İstatistik Kurumu Verileriyle Veri Zarflama Analizi’ne Dayalı Bir Endeks. Eskişehir Osmangazi Üniversitesi İktisadi ve İdari Bilimler Dergisi.

[4] Atölyesi K, Virüs Mü, Yoksulluk Mu (2020). Korona Virüs Salgınının Mevsimlik Gezici Tarım İşçileri Ve Onların Çocukları İle Bitkisel Üretime Olası Etkisi Hızlı Bir Değerlendirme.

[5] Oecd. How’s Life? Measuring Well-Being. Paris: Oecd Publishing; 2011. https://www.oecd.org/wise/how-s-life-23089679.htm. Acsessed 10 Dec 2022.

[6] Allin P (2007). Measuring Societal Wellbeing. Economic&Labour Market Review.

[7] Ertaş A, Kağan G, Akçi Y, Zelka M (2021). Türk Toplumunun Covid-19’a İlişkin Bilgi Tutum Ve Uygulamaları. Ekev Akademi Dergisi.

[8] Gruber E, Vukovic IS, Musovic M, Moravek D, Starcevic B, Martic-Biocina S, Knez R (2020). Personal wellbeing, work ability, satisfaction with life and work in psychiatrists who emigrated from Croatia. Psychiatr Danub.

[9] Lotfy NA (2020). Validity And reliability of the arabic version of the" personal wellbeing index-adults" on adults with hearing impairment. Health Promot Perspect.

[10] Weerasinghe S. "Occupational Health and Safety Standards of Foreign Seasonal Farm Workers: Evaluation of Personal Protection Measures, Policies and Practices", in Occupational Wellbeing. London: IntechOpen; 2020. [Online]. Available: https://www.intechopen.com/chapters/73571, 10.5772/intechopen.94056.

[11] Winefield HR, Gill TK, Taylor AW, Pilkington RM (2012). Psychological well-being and psychological distress: is it necessary to measure both?. Psychol Well-Being Theory Res Pract.

[12] Ross KL, Zereyesus YA, Shanoyan A, Amanor-Boadu V (2015). The health effects of women empowerment: recent evidence from northern Ghana. Int Food Agribusiness Manag Rev.

[13] Davey G, Chen Z, Lau A (2009). Piece in a thatched hut-that is hapiness: subjective wellbeing among peasants in rural china. J Hapiness Study.

[14] Degutis M, Ve US (2013). Litvanya'da Öznel İyi Oluşun Belirleyicileri. Mühendislik Ekonomisi.

[15] Palomino PJ (2019). Regional Well-Being İn The Oecd. J Econ Inequality.

[16] Knight J, Song L, Gunatilaka R. Subjective well-being and its determinants in rural China. China economic review. 2009;20(4):635–49.

[17] Sun S (2016). Subjective well-being and its association with subjective health status, age, sex, region, and socio-economic characteristics in a Chinese population study. J Happiness Stud..

[18] Schirmer J, Berry HL, O’Brien LV (2013). Healthier land, healthier farmers: considering the potential of natural resource management as a place-focused farmer health intervention. Health Place.

[19] Brew B (2016). The health and wellbeing of Australian farmers: a longitudinal cohort study.". BMC Public Health.

[20] Vosko LF (2022). Understanding migrant farmworkers’ health and well-being during the Global COVID-19 pandemic in Canada: toward a transnational conceptualization of employment strain. Int J Environ Res Public Health.

[21] Kaliterna LJ, Prizmic-Larsen Z, Michalos AC (2014). Personal Wellbeing Index İn Croatia. Encyclopedia Of Quality Of Life And Well-Being Research.

[22] Biddle N, Edwards B, Gray M, Sollis K (2020). Hardship, distress, and resilience: the initial impacts Of Covıd-19 İn Australia.

[23] İnel A, Derya AT, Coşkun E, Bozkurt A (2021). Yaşlılarda Covıd-19 Pandemi Sürecinde Bilinçli Farkındalık, Başa Çıkma Tutumları Ve Kişisel İyi Oluş. Turk J Fam Med Prim Care.

[24] Tomar BS, Singh P, Nathiya D, Suman S, Raj P, Tripathi S, Chauhan DS (2021). Indian community's knowledge, attitude, and practice toward Covıd-19. Indian J Soc Psychiatry.

[25] Kılıç M, Uslukılıç G, Ok Ş (2021). Pandemisi evde kal uygulaması: Toplumun tutum ve davranışları (the stay at home isolation for Covid-19 pandemic: attitude and behavior of the society). Bozok Tıp Dergisi.

[26] Şirin H, Ketrez G, Ahmadi AA, Arslan A, Altunel E, Güneş İS, Hasde M (2020). Türkiye'de Covıd-19'a Yönelik Toplum Yaklaşımı: İlk Vaka Görüldükten Bir Ay Sonra. Türk Hijyen Ve Deneysel Biyoloji Dergisi.

[27] Reuben RC, Danladi MM, Saleh DA, Ve Ejembi PE (2021). Covıd-19'a Yönelik Bilgi, Tutum Ve Uygulamalar: Kuzey-Orta Nijerya'da Epidemiyolojik Bir Araştırma. Toplum Sağlığı Dergisi.

[28] Quandt SA (2020). COVID-19 pandemic among Latinx farmworker and nonfarmworker families in North Carolina: knowledge, risk perceptions, and preventive behaviors. Int J Environ Res Public Health.

[29] Alıcılar HE, Güneş G, Meltem ÇÖ (2020). Toplumda Covıd-19 Pandemisiyle İlgili Farkındalık, Tutum Ve Davranışların Değerlendirilmesi. Estüdam Halk Sağlığı Dergisi.

[30] Abdelhafiz AS, Mohammed Z, Ibrahim ME, Ziady HH, Alorabi M, Ayyad M, Vd. Knowledge, Perceptions, And Attitude Of Egyptians Towards The Novel Coronavirus Disease (Covıd-19). J Community Health. 2020;45(5):1–10.14. 10.1007/s10900-020-00827-7 PMC7173684 32318986

[31] WOLF, Michael S, et al. Awareness, attitudes, and actions related to COVID-19 among adults with chronic conditions at the onset of the US outbreak: a cross-sectional survey. Annals of internal medicine. 2020;173(2):100–09. 10.7326/M20-1239 PMC7151355 32271861

[32] Chen Y, Jin YL, Zhu LJ, Fang ZM, Wu N, Du MX (2020). The network investigation on knowledge, attitude and practice about Covıd-19 Of the residents İn Anhui Province. Zhonghua Yu Fang Yi Xue Za Zhi.

[33] Zhong BL, Luo W, Li HM, Zhang QQ, Liu XG, Li W-T (2020). Knowledge, attitudes, and practices towards covid-19 among Chinese residents during the rapid rise period of the Covıd-19 outbreak: a quick online cross-sectional survey. Int J Biol Sci.

[34] Tanır F. Tarımda İş Sağlığı ve Güvenliği. 2016. Date: 10.04.2022 Available: http://safetyhealth.com.tr/occupational-health-and-safety-in-agriculture/.

